# Supplementary material for: Fibroblast growth factor signaling induces a chondrocyte-like state of peripheral nerve fibroblast during aging
Source: Nat Commun. 2025 Nov 14;16:10020. doi: 10.1038/s41467-025-65297-8 (PMC12618493; doi:10.1038/s41467-025-65297-8)
Supplement: Supplementary file 1 — Supplementary Information [file 41467_2025_65297_MOESM1_ESM.pdf]

Title: Fibroblast Growth Factor signaling induces a chondrocyte-like state of peripheral nerve fibroblast during aging

Authors: Dragana Stefanovska[1,2], Eliza Sassu[1], Mehmet Tekman[1], Amirhossein Naghsh Nilchi[1,3], Severin Haider[1], Claudia Domisch[1], Madelon Hossfeld[1], Stefanie Perez-Feliz[2], Lauritz Miarka[4], Franziska Schneider-Warme[2,5], Sebastian J. Arnold[1,5], Marco Prinz[4,5,6], Björn Grüning[3], Sebastian Preissl*[1,5,7,8], Luis Hortells*[1,9]

[1] Institute of Experimental and Clinical Pharmacology and Toxicology, Faculty of Medicine, University of Freiburg, 79104 Freiburg, Germany.
[2] Institute for Experimental Cardiovascular Medicine, University Heart Center Freiburg · Bad Krozingen, University of Freiburg, 79110 Freiburg, Germany.
[3] Bioinformatics Group, Department of Computer Science, Albert-Ludwigs-University Freiburg, Freiburg, Germany
[4] Institute of Neuropathology, Faculty of Medicine, University of Freiburg, 79106 Freiburg, Germany
[5] CIBSS – Centre for Integrative Biological Signalling Studies, University of Freiburg, 79104 Freiburg, Germany.
[6] Center Brain Research and Advancements In Neuroimmunology (BRAIN), Faculty of Medicine, University of Freiburg, 79106 Freiburg, Germany
[7] Institute of Pharmaceutical Sciences, Pharmacology & Toxicology, University of Graz, 8010 Graz, Austria
[8] Field of Excellence BioHealth, University of Graz, Graz, Austria.
[9] Cardiovascular Research Group, Department of Medical Biology, Faculty of Health Science, UiT-The Arctic University of Norway, 9019 Tromsø, Norway.
* Corresponding author

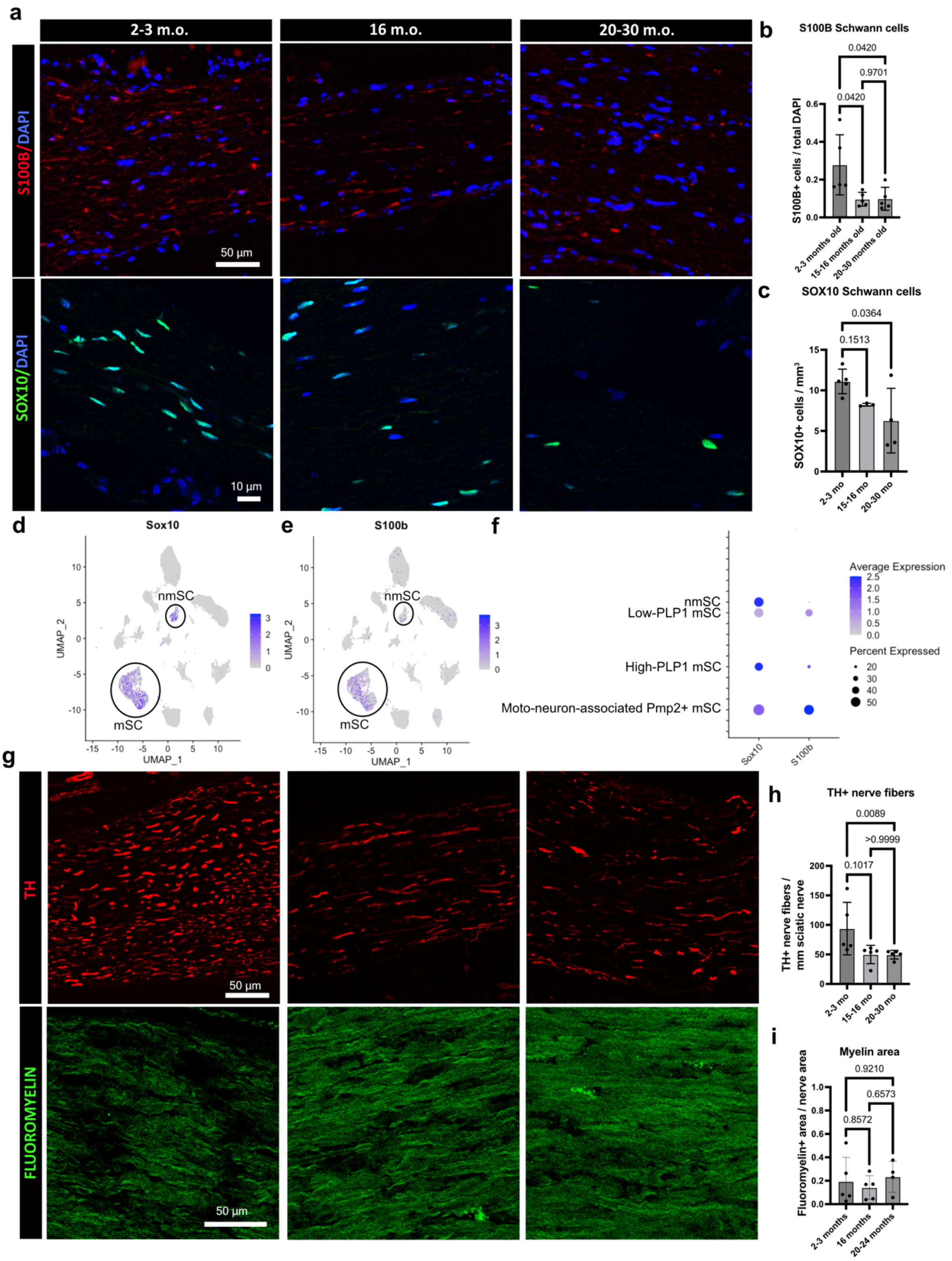

Figure S1: Representative immunofluorescence images of sciatic nerve samples show **a** less S100B+ Schwann cells in sciatic nerves of 15-16 months mice and less SOX10+ Schwann cells in nerves from 20-30 months old mice. **b** Quantification of the relative presence of S100B+ Schwann cells. n = 5, biological replicates. **c** Quantification of the relative presence of SOX10+ Schwann cells. n = 5 - 3 - 4, biological replicates. **d** First level clustering UMAP shows *Sox10* expression in all the Schwann cell clusters. **e** First level clustering UMAP shows *S100b* expression in all the myelinating Schwann cell clusters but not in the non-myelinating Schwann cell cluster. **f** Dot plot of *Sox10* and *S100b* expression in first level clusters present in sciatic nerves from mice of all ages. Sox10 is expressed in all the Schwann cell populations while S100b is manly expressed in the myelinating Schwann cell populations. **g** Representative immunofluorescence images of sciatic nerve samples show less TH+ nerve fibers in sciatic nerves from 20-30 months old, and unchanged myelin area in sciatic nerves across time points. **h**

Quantification of the number of TH+ nerve fibers rationalized by nerve thickness. n = 5, biological replicates. **i** Quantification of the area of fluoromyelin rationalized by nerve area. n = 5, biological replicates. Data are presented as mean values +/- SD. Source data for panels is provided as a Source Data file.

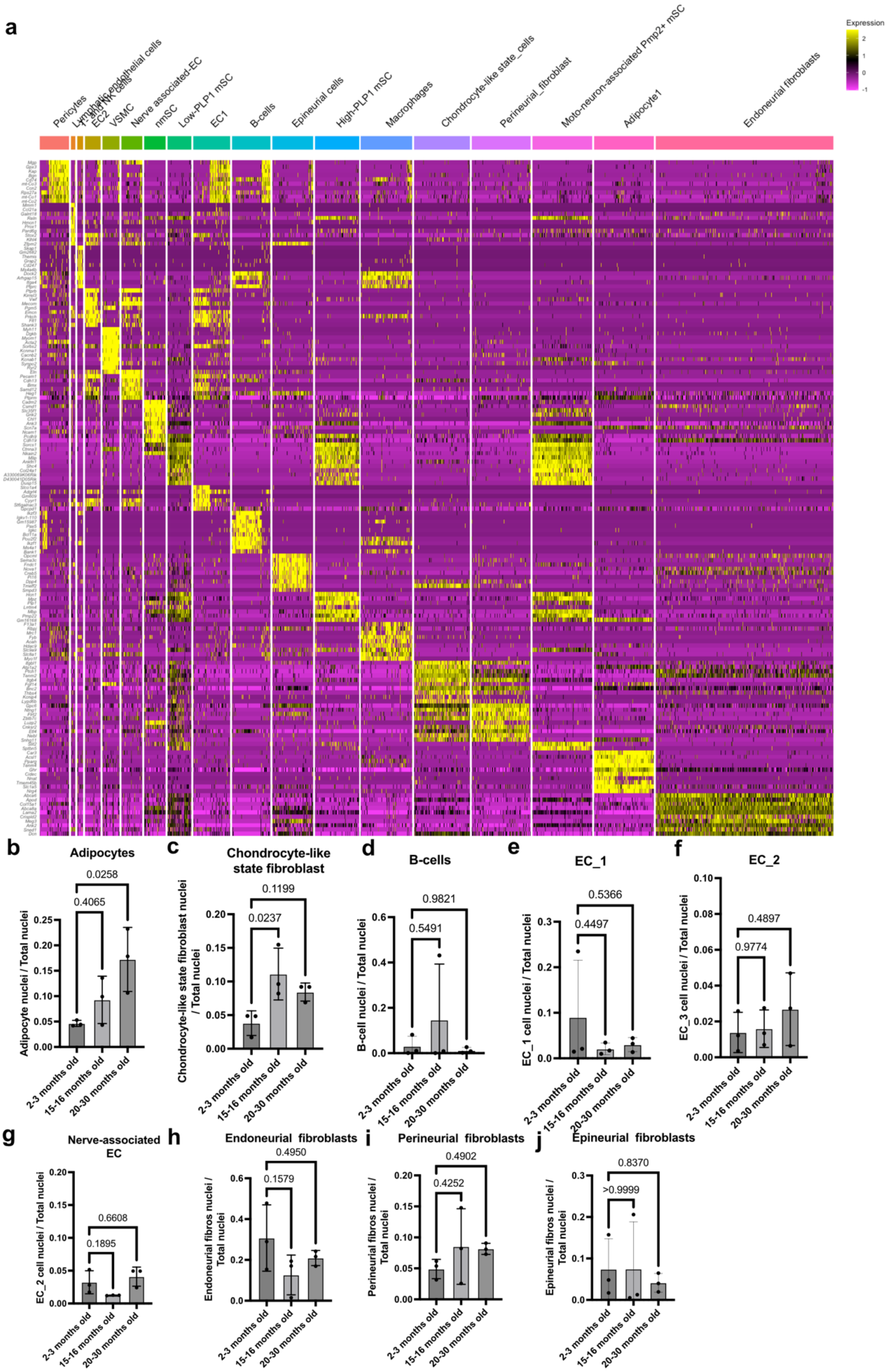

Figure S2. Adipocyte and chondrocyte-like state fibroblast density are increased in sciatic nerves during aging. a Heatmap of the top 10 differentially expressed genes by cluster. Number of nuclei of adipocytes **b**, chondrocyte-like state fibroblasts **c**, B-cells **d**, EC_1 **e**, EC_2 **f**, nerve-associated EC **g**, endoneurial fibroblasts **h**, perineurial fibroblastc **i**, and epineurial fibroblasts **j** in relation to the total number of nuclei for each animal studied. Significantly more chondrocyte-like cells were detected in the nerves from 15-16- and 20-30-months old mice, while adipocytes density was significantly higher only in the 20-30 months old group. No significant differences were

observed in B-cells or endothelial cells. n = 3, biological replicates. Data are presented as mean values +/- SD. Source data for panels is provided as a Source Data file.

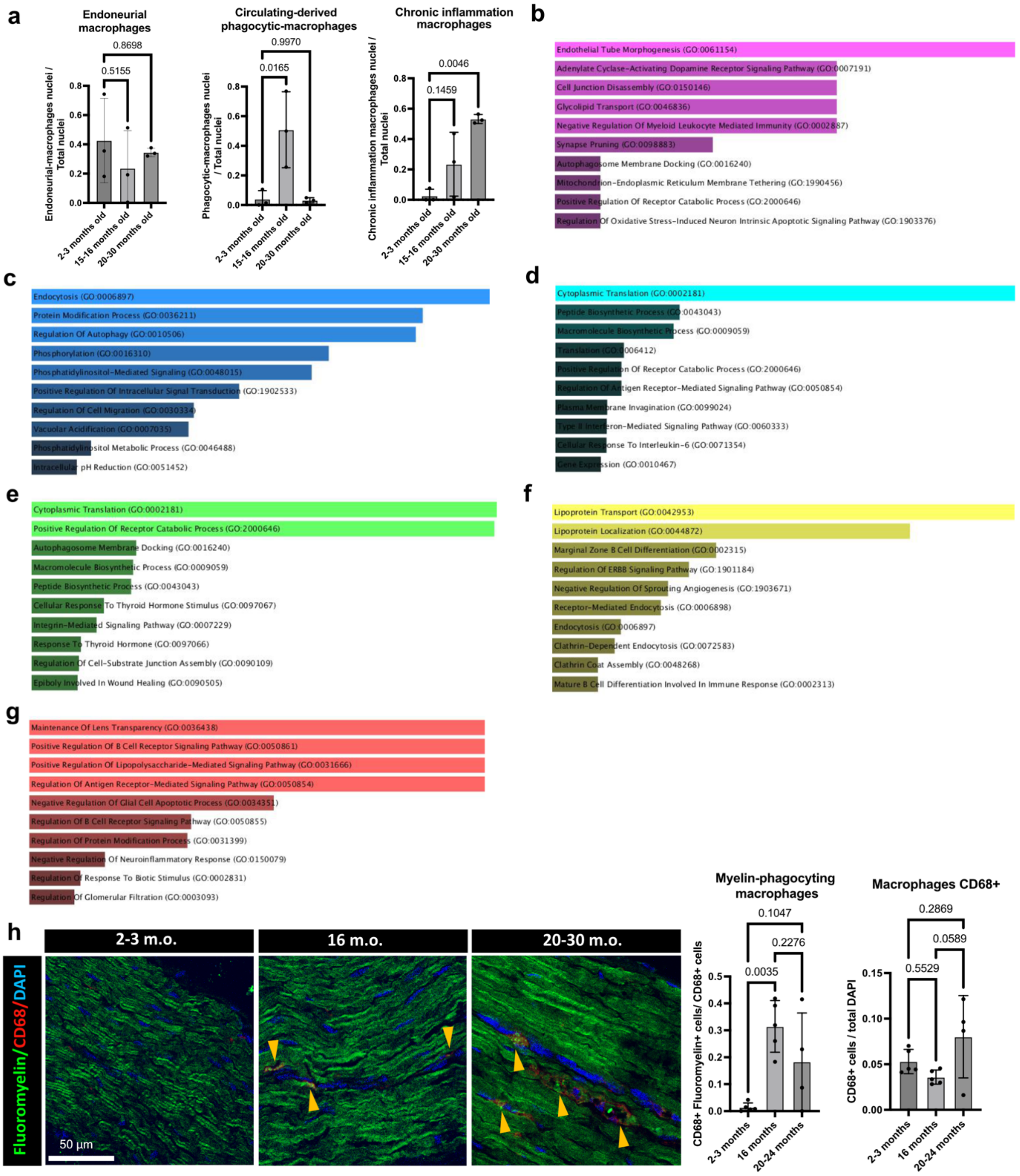

Figure S3. Mϕs enrichment and biological function of sciatic nerves. a Number of nuclei of M2, phagocytic, and M1 Mϕs sub-cluster in relation to the total number of Mϕs for each animal studied. Phagocytic-Mϕs and M1-Mϕs numbers were increased in the nerves from 15-16 months and 20-30 months-old mice, respectively. Gene Ontotology Biological Function based on differentially expressed genes of **b** Endoneurial macrophages - Mϕs **c** Chronic ifnlammation - Mϕs **d** Circulating Mϕs_2 **e** Phagocytic - Mϕs **f** Epineurial - Mϕs **g** Circulating Mϕs_1. n = 3, biological replicates. **h** Representative immunofluorescence images of sciatic nerve samples show increased CD68+ macrophages with phagocyted myelin in sciatic nerves from 15-16 m.o. mice, compared to 2-3 m.o. mice. No statistically significant change was observed in the relative number of CD68+ macrophages. Yellow arrows point to CD68+/Fluoromyelin+ macrophages. n = 5 - 5 – 4, biological replicates. Data are presented as mean values +/- SD. Source data for panels is provided as a Source Data file.

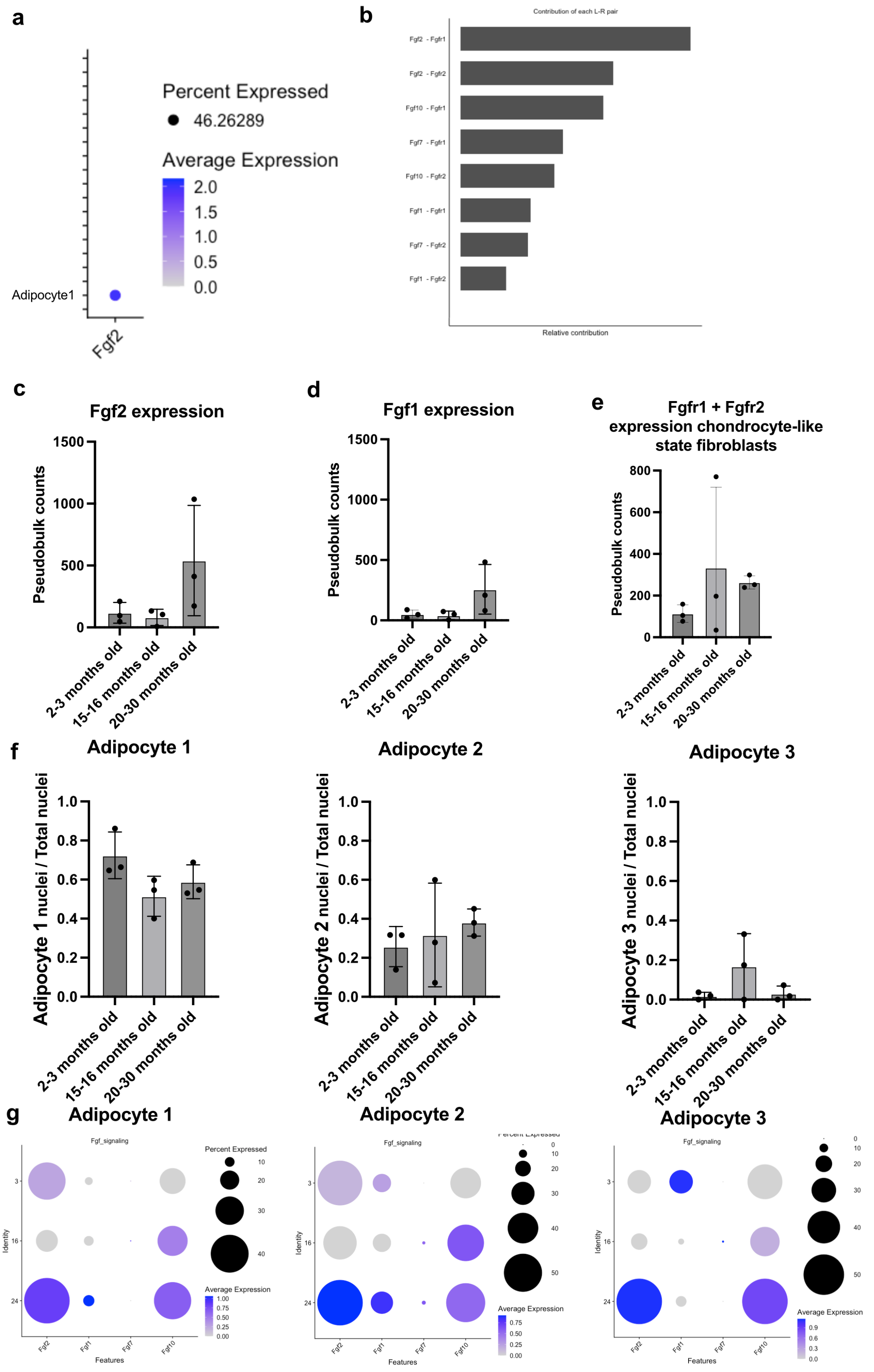

Figure S4: a Dot plot of *Fgf2* expression in first level clusters present in sciatic nerves from mice of all ages. Higher expression of Fgf2 was observed in cluster 2, which corresponds with adipocyte cluster. **b** Cell-Chat predicted contribution of each ligand-receptor of the FGF pathway in a possible crosstalk between adipocytes and neural fibroblasts. The top scores predicted were *Fgf2-Fgfr1* and *Fgf2-Fgfr2*. **c**, **d** Pseudobulk raw counts of *Fgf2* **c** and *Fgf1* **d** in adipocytes from sciatic nerves from mice of different ages. n = 3, biological replicates. **e** Pseudobulk raw counts of *Fgfr1* and *Fgfr2* in chondrocyte-like state fibroblasts from sciatic nerves of mice of different ages. n = 3 **f** Number of nuclei of each adipocyte sub-cluster in relation to the total number of adipocytes for each animal studied.

No significant differences were found for any sub-cluster at any age point. n = 3, biological replicates**. g** Dot plot of *Fgf2, Fgf1, Fgf7, and Fgf10* expression in second level clusters of adipocytes present in sciatic nerves from mice of all ages. Data are presented as mean values +/- SD. Source data for panels is provided as a Source Data file.

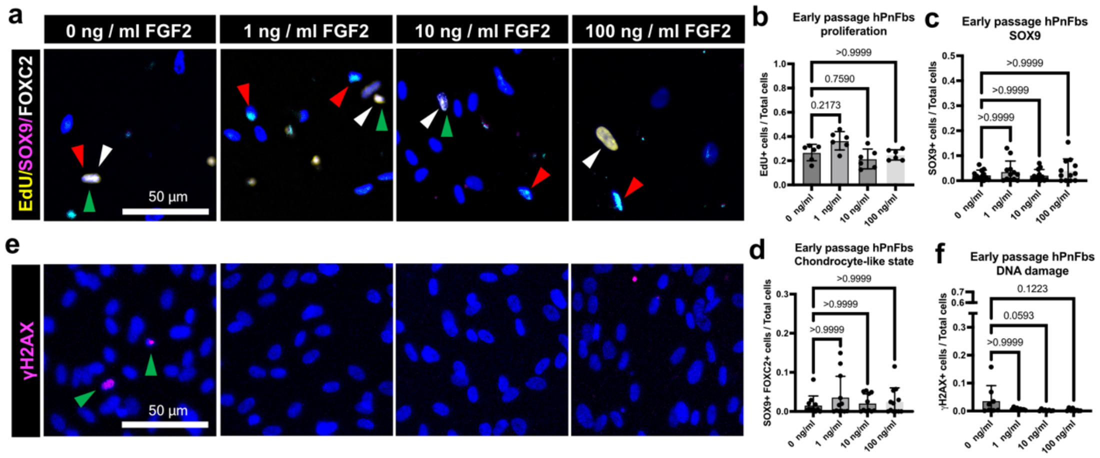

Figure S5. FGF2 reduces DNA damage of *in vitro* hPnFbs early passages. **a** Representative immunocytochemistry images of early passage hPnFbs exposed to different concentrations of FGF2. White arrows point to EdU+ nuclei, green arrows point to SOX9+ cells, red arrows point to FOXC2+ cells. **b** Quantification of the density of EdU+ nuclei. n = 6, biological replicates. **c** Quantification of the density of SOX9+ nuclei. n = 12, biological replicates. **d** Quantification of the density of SOX9+/FOXC2+ nuclei. n = 12, biological replicates. **e** Representative immunocytochemistry images of early passage hPnFbs exposed to different concentrations of FGF2. Green arrows point to γH2AX+ nuclei **f** Quantification of the density of γH2AX+. n = 8, biological replicates. Data are presented as mean values +/- SD. Source data for panels is provided as a Source Data file.

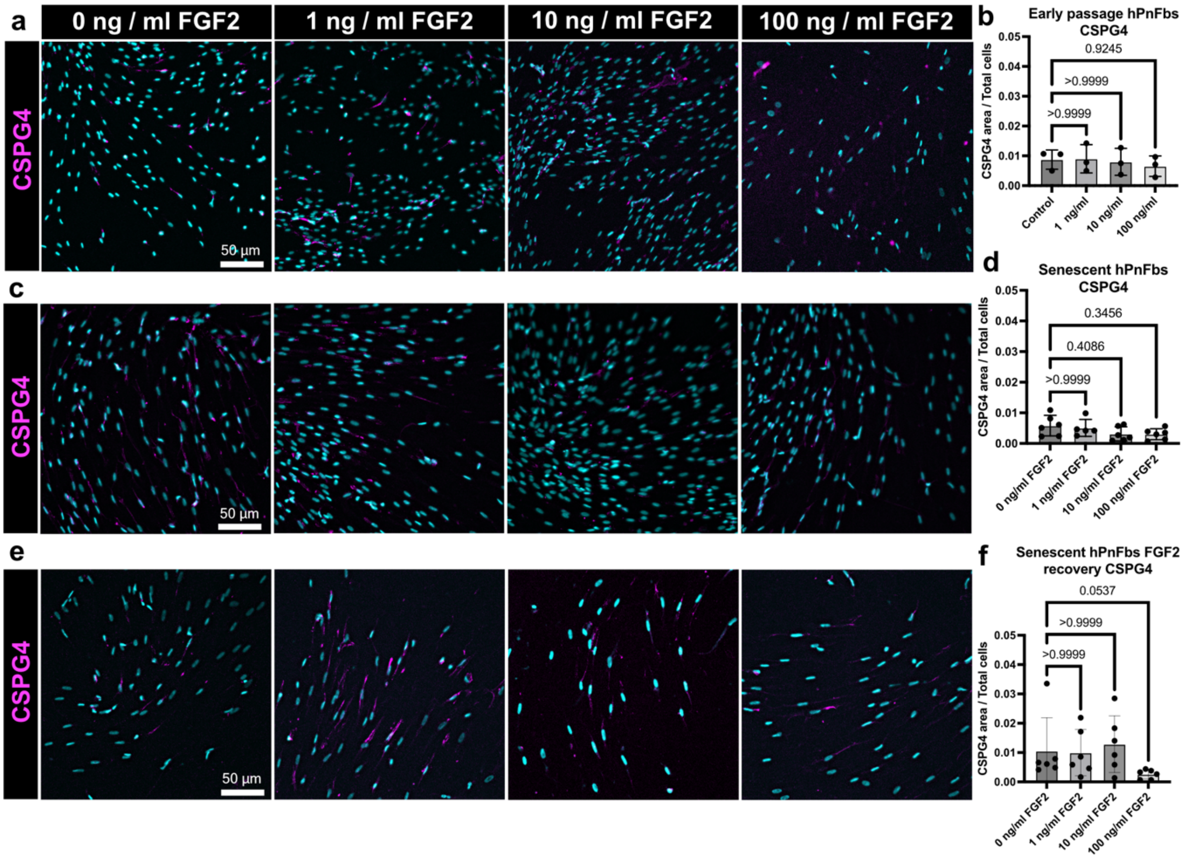

Figure S6. *In vitro,* FGF2 does not increase CSPG4 expression in hPnFbs. a Representative immunocytochemistry images of early passage hPnFbs exposed to different concentrations of FGF2. **b** Quantification of the area of CSPG4 by total cell number. n = 3, biological replicates. **c** Representative immunocytochemistry images of senescent hPnFbs exposed to different concentrations of FGF2. **d** Quantification of the area of CSPG4 by total cell number. n = 6, biological replicates. **e** Representative immunocytochemistry images of senescent hPnFbs exposed to different concentrations of FGF2 for 14 days, with a posterior withdrawal for 7 days. Significantly less CSPG4 was detected after withdrawal of the highest dose of FGF2 (100 ng/ml) **f** Quantification of the area of CSPG4 by total cell number with the 7 days withdrawal. n = 6, biological replicates. Data are presented as mean values +/- SD. Source data for panels is provided as a Source Data file.

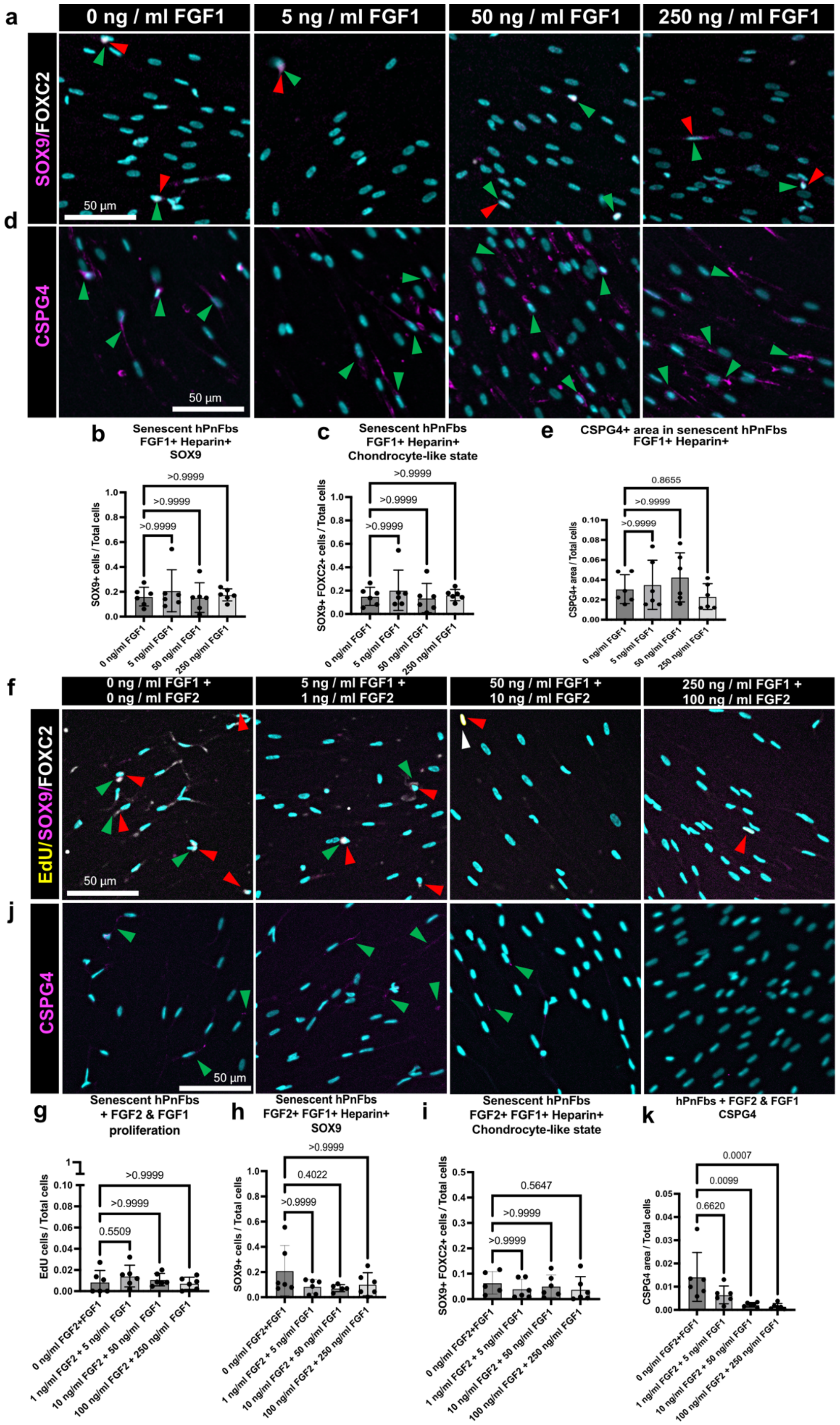

Figure S7. FGF1 blocks FGF2 activation of SOX9+ FOXC2+ co-expression and the expression of CSPG4 in senescent hPnFbs *in vitro*. a Representative immunocytochemistry images of senescent hPnFbs exposed to heparin and different concentrations of FGF1. Green arrows point to SOX9+ cells, red arrows point to FOXC2+ cells. **b** Quantification of the density of SOX9+ nuclei. n = 6, biological replicates. **c** Quantification of the density of SOX9+/FOXC2+ nuclei. n = 6, biological replicates. **d** Representative immunocytochemistry images of senescent hPnFbs exposed to heparin and different concentrations of FGF1. **e** Quantification of the area of CSPG4 by total

cell number. n = 6, biological replicates. **f** Representative immunocytochemistry images of senescent hPnFbs exposed to heparin and different concentrations of FGF1 and FGF2. White arrows point to EdU+ nuclei, green arrows point to SOX9+ cells, red arrows point to FOXC2+ cells. **g** Quantification of the density of EdU+ nuclei. **h** Quantification of the density of SOX9+ nuclei. n = 6, biological replicates. **i** Quantification of the density of SOX9+/FOXC2+ nuclei. n = 6. **j** Representative immunocytochemistry images of senescent hPnFbs exposed to heparin different concentrations of FGF1 and FGF2. **k** Quantification of the area of CSPG4 by total cell number. n = 6, biological replicates. Data are presented as mean values +/- SD. Source data for panels is provided as a Source Data file.

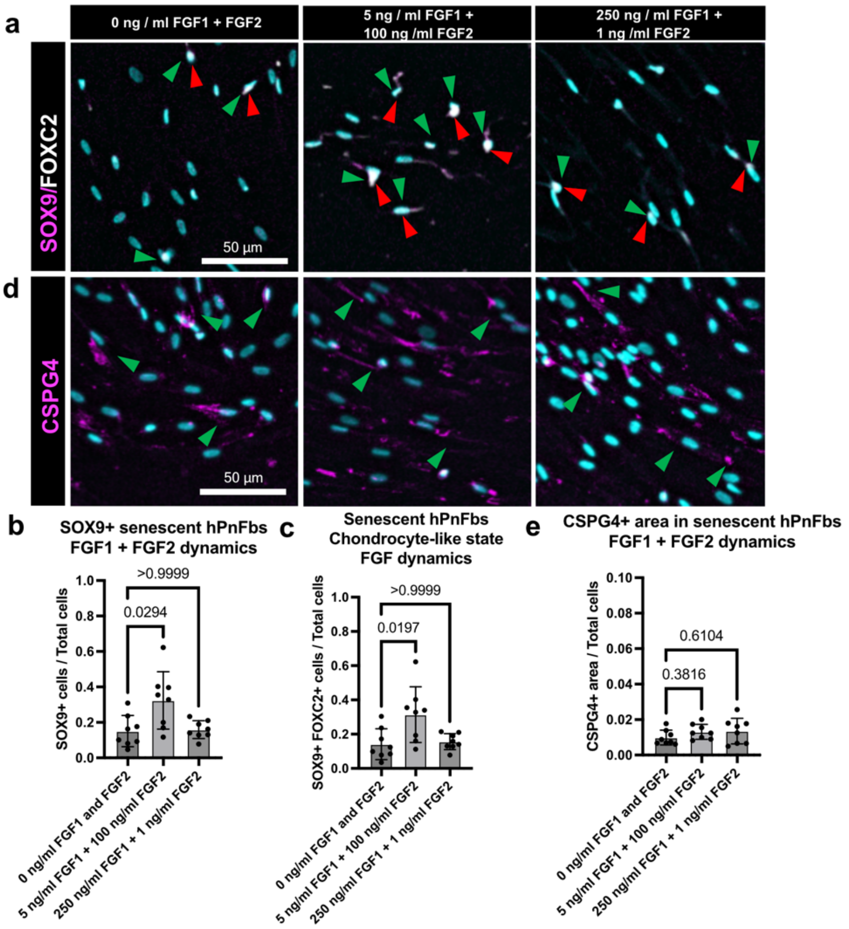

Figure S8. Lower concentration of FGF1 does not block FGF2 activation of a chondrocyte-like state in senescent hPnFbs *in vitro*. a Representative immunocytochemistry images of senescent hPnFbs exposed to heparin and different concentrations of FGF1 and FGF2. Red arrows point to SOX9+ cells, green arrows point to FOXC2+ cells. **b** Quantification of the density of SOX9+ nuclei. n = 8. **c** Quantification of the density of SOX9+/FOXC2+ nuclei. n = 8. **d** Representative immunocytochemistry images of senescent hPnFbs exposed to heparin and different concentrations of FGF1 and FGF2. **e** Quantification of the area of CSPG4 by total cell number. n = 8. Data are presented as mean values +/- SD. Source data for panels is provided as a Source Data file.
