# Supplementary material for: Fibroblast growth factor signaling induces a chondrocyte-like state of peripheral nerve fibroblast during aging
Source: Nat Commun. 2025 Nov 14;16:10020. doi: 10.1038/s41467-025-65297-8 (PMC12618493; doi:10.1038/s41467-025-65297-8)
Supplement: Supplementary file 2 — Reporting Summary [file 41467_2025_65297_MOESM2_ESM.pdf]

Corresponding author(s): Luis Hortells, Sebastian Preissl

Last updated by author(s): 2025-09-04

Reporting Summary

Nature Portfolio wishes to improve the reproducibility of the work that we publish. This form provides structure for consistency and transparency in reporting. For further information on Nature Portfolio policies, see our Editorial Policies and the Editorial Policy Checklist.

Statistics

For all statistical analyses, confirm that the following items are present in the figure legend, table legend, main text, or Methods section.

n/a	Confirmed	
☐	☒	The exact sample size (*n*) for each experimental group/condition, given as a discrete number and unit of measurement
☐	☒	A statement on whether measurements were taken from distinct samples or whether the same sample was measured repeatedly
☐	☒	The statistical test(s) used AND whether they are one- or two-sided<br>*Only common tests should be described solely by name; describe more complex techniques in the Methods section.*
☒	☐	A description of all covariates tested
☒	☐	A description of any assumptions or corrections, such as tests of normality and adjustment for multiple comparisons
☐	☒	A full description of the statistical parameters including central tendency (e.g. means) or other basic estimates (e.g. regression coefficient) AND variation (e.g. standard deviation) or associated estimates of uncertainty (e.g. confidence intervals)
☐	☒	For null hypothesis testing, the test statistic (e.g. *F*, *t*, *r*) with confidence intervals, effect sizes, degrees of freedom and *P* value noted<br>*Give P values as exact values whenever suitable.*
☒	☐	For Bayesian analysis, information on the choice of priors and Markov chain Monte Carlo settings
☒	☐	For hierarchical and complex designs, identification of the appropriate level for tests and full reporting of outcomes
☒	☐	Estimates of effect sizes (e.g. Cohen's *d*, Pearson's *r*), indicating how they were calculated

Our web collection on statistics for biologists contains articles on many of the points above.

Software and code

Policy information about availability of computer code

Data collection	Leica Application Suite (3.7.5.24914)
Data analysis	Rstudio (2023.03.0+386), Fiji Image J (2.14.0/1.54f), GraphPad Prism (9.5.1), CellRanger (7.0.0, 10x Genomics), Harmony (0.1.1), Seurat (v4.3.0.1), CellChat (v1.6.1), Monocle (v1.3.1), Doublet Finder (v2.0.3))

For manuscripts utilizing custom algorithms or software that are central to the research but not yet described in published literature, software must be made available to editors and reviewers. We strongly encourage code deposition in a community repository (e.g. GitHub). See the Nature Portfolio guidelines for submitting code & software for further information.

Data

Policy information about availability of data

All manuscripts must include a data availability statement. This statement should provide the following information, where applicable:

- Accession codes, unique identifiers, or web links for publicly available datasets
- A description of any restrictions on data availability
- For clinical datasets or third party data, please ensure that the statement adheres to our policy

To facilitate the visualization and exploration of the single-nucleus RNA-seq data, we developed a docker (docker.com) container to run a CELLxGENE instance. This container includes the complete snRNA-seq dataset and the second-level Adipocyte and Macrophage clustering. To enhance accessibility, this container was integrated to the European Galaxy server (usegalaxy.eu) as an interactive tool. This tool launches CELLxGENE with the selected dataset and provides a web-based interface for visualization (https://usegalaxy.eu/?tool_id=interactive_tool_cellxgene_mouse_sciatic_nerve&version=latest)

Raw sequencing data is deposited to GEO under accession number GSE280857 (https://www.ncbi.nlm.nih.gov/geo/query/acc.cgi?acc=GSE280857). Processed data including UMAPs from first and second level clustering are deposited on Zenodo (https://doi.org/10.5281/zenodo.14900774).
Source data for quantification of additional data is provided in a Source Data file.

Research involving human participants, their data, or biological material

Policy information about studies with human participants or human data. See also policy information about sex, gender (identity/presentation), and sexual orientation and race, ethnicity and racism.

Reporting on sex and gender	Not reported, data protection
Reporting on race, ethnicity, or other socially relevant groupings	Not reported, data protection
Population characteristics	Age of human donors:<br>Nervus accesorius (17 years old), nervus suralis (24, 25, 31, 55, and 63 years old), nervus femoralis (33 years old), nervus hypoglossus (57 and 68 years old) and nervus ulnaris (65 years old)
Recruitment	No human individuals were recruited for this study. Tissue sections for staining were generated from already existing FFPE tissue blocks
Ethics oversight	FFPE-nerve tissue were generated from surgical specimens in accordance with an Institutional Review Board–approved protocol (Ethic Commission of Albert-Ludwigs University of Freiburg: 1008/09).

Note that full information on the approval of the study protocol must also be provided in the manuscript.

Field-specific reporting

Please select the one below that is the best fit for your research. If you are not sure, read the appropriate sections before making your selection.

☒ Life sciences ☐ Behavioural & social sciences ☐ Ecological, evolutionary & environmental sciences

For a reference copy of the document with all sections, see nature.com/documents/nr-reporting-summary-flat.pdf

Life sciences study design

All studies must disclose on these points even when the disclosure is negative.

Sample size	Sample sizes were determined based on assay throughput and published work on similar subjects.
Data exclusions	For downstream analysis of snRNA-seq only nuclei with 800 to 7,000 detected genes and less than 5% mitochondrial RNA were included. Doublets were removed using DoubletFinder, 5 clusters with markers from multiple cell types and two residual ekeltal muscle cell clustres were also removed before downstream analysis.<br>No data were exluded from the other analyses (histology, in vitro)
Replication	Individual snRNA-seq libraries were generated for sciatic nerve nuclei from 3 mice per age group. Both sciatic nerves from a single mouse were pooled for library preparation. For the in vitro studies, each experiment was repeated at least four times.<br>5 human tissue samples were analyzed per age group. Sections from 3-10 mice were analyzed per age group in mice.
Randomization	Samples were allocated based on age
Blinding	Investigators were not blinded

Reporting for specific materials, systems and methods

We require information from authors about some types of materials, experimental systems and methods used in many studies. Here, indicate whether each material, system or method listed is relevant to your study. If you are not sure if a list item applies to your research, read the appropriate section before selecting a response.

Materials & experimental systems

n/a	Involved in the study	
☐	☒	Antibodies
☐	☒	Eukaryotic cell lines
☒	☐	Palaeontology and archaeology
☐	☒	Animals and other organisms
☒	☐	Clinical data
☒	☐	Dual use research of concern
☒	☐	Plants

Methods

n/a	Involved in the study	
☒	☐	ChIP-seq
☐	☒	Flow cytometry
☒	☐	MRI-based neuroimaging

Antibodies

Antibodies used	Target antigen, Order number, Provider<br>CD45, AF114, R&D systems<br>CD68 MCA1957, Bio-Rad<br>CSPG4 ab129051, Abcam<br>FABP4 ab92501, Abcam<br>FGF2 MA5-15276, Invitrogen<br>FOXC2 AF6989, Novus biologicals<br>γH2AX 9718, Cell Signaling Technology<br>GLUT1/SLC2A1, ab150299, Abcam<br>GRN, ab187070, Abcam<br>KCNC2, PA5-36072, Invitrogen<br>MRC1, ab64693, Abcam<br>SOX10, ab180862, Abcam<br>S100B, ab52642, Abcam<br>SOX9, ab185966, Abcam<br>SOX9, AF3075, R&D systems<br>TGFBR1, 30117-1-AP, Proteintech<br>TH, ab137869, Abcam<br>VIMENTIN, ab20346, Abcam
Validation	Validation of antibodies is performed by the manufacturer. In addition, an initial working concentration test was performed, including using a "no-primary antibody" negative control.

Eukaryotic cell lines

Policy information about cell lines and Sex and Gender in Research

Cell line source(s)	Primary hPnFbs were purchased from ScienCell Research Laboratories (1710, Carlsbad, CA, USA)
Authentication	Cells were not authenticated
Mycoplasma contamination	Cells were not tested for mycoplasma contamination
Commonly misidentified lines<br>(See ICLAC register)	Does not apply

Animals and other research organisms

Policy information about studies involving animals; ARRIVE guidelines recommended for reporting animal research, and Sex and Gender in Research

Laboratory animals	Sox10-CreERT2 (Sox10CreERT2, Jax reference: 009074) mice were bred with R26tdt/tdt mice (Jax reference: 027651) mice to genetically trace Schwann cells. Experiments were performed on tissue obtained from young-adult (age: 2–3 months) adult (15-16 months) or old (age: 20–30 months) mice.
Wild animals	This study did not involve wild animals
Reporting on sex	Mice from both sexes were included indistinctively in this study
Field-collected samples	This study did not involve field-collected samples
Ethics oversight	All animal procedures were carried out in accordance with the guidelines stated in Directive 2010/63/EU of the European Parliament and of the Council of 22 September 2010 and were approved by the local authorities (Regierungspräsidium Freiburg; approval numbers X23-11R and G22-090).

Note that full information on the approval of the study protocol must also be provided in the manuscript.

Plants

Seed stocks	*Report on the source of all seed stocks or other plant material used. If applicable, state the seed stock centre and catalogue number. If plant specimens were collected from the field, describe the collection location, date and sampling procedures.*
Novel plant genotypes	*Describe the methods by which all novel plant genotypes were produced. This includes those generated by transgenic approaches, gene editing, chemical/radiation-based mutagenesis and hybridization. For transgenic lines, describe the transformation method, the number of independent lines analyzed and the generation upon which experiments were performed. For gene-edited lines, describe the editor used, the endogenous sequence targeted for editing, the targeting guide RNA sequence (if applicable) and how the editor was applied.*
Authentication	*Describe any authentication procedures for each seed stock used or novel genotype generated. Describe any experiments used to assess the effect of a mutation and, where applicable, how potential secondary effects (e.g. second site T-DNA insertions, mosiacism, off-target gene editing) were examined.*

Flow Cytometry

Plots

Confirm that:

- [ ] The axis labels state the marker and fluorochrome used (e.g. CD4-FITC).
- [ ] The axis scales are clearly visible. Include numbers along axes only for bottom left plot of group (a 'group' is an analysis of identical markers).
- [ ] All plots are contour plots with outliers or pseudocolor plots.
- [ ] A numerical value for number of cells or percentage (with statistics) is provided.

Methodology

Sample preparation

From each mouse, both snap frozen femoral sciatic nerves were thawed in nuclei isolation media (NIM, 250 mM Sucrose, 25 mM KCl, 5 mM MgCl2, 10 mM Tris-HCl) with 25 mM DTT, 1X protease inhibitor (05056489001, Roche), 0.1% Triton X-100 and RNase inhibitor (N2515, Promega). Each pair of nerves was homogenized using a Kimble dounce tissue grinder (D8938, Merck) and the resulting suspension was filtered through a 30 µm CellTRics (Sysmex, MSA150914) into a LoBind Eppendorf tube (Eppendorf). Nuclei were pelleted (1000 x g, 10 min, 4oC) and washed with NIM + DTT + Protease inhibitor (05056489001, Roche) and RNase inhibitor (N2515, Promega). After the wash, nuclei were resuspended in sorting buffer (1 mM EDTA, 0.2 U/µL RNase inhibitor (N2515, Promega), 2% fatty acid-free BSA (A7030-100g, Sigma) in PBS). Finally, the nuclei were filtered again through a 30 µm CellTrics (Sysmex, MSA150914), and DRAQ7 (#7406, Cell Signalling) was added to the nuclei suspension for nuclei detection by flow cytomtery.

Instrument

10,000-20,000 nuclei were sorted using a S3 Fluorescence-activated cell sorter (Bio-Rad).

Software

ProSort™ Software v1.6

Cell population abundance

Does not apply.

Gating strategy

Nuclei were pooled and stained with DRAQ7. First, potential nuclei were identified using forward scatter (FSC) area and back scatter (BSC) area. Next, potential doublets were removed based on BSC and FSC signal width. Nuclei were sorted based on DRAQ7 high signal.

- [ ] Tick this box to confirm that a figure exemplifying the gating strategy is provided in the Supplementary Information.
